# Utilizing Video-Based Trainings to Improve Decision Making in High School Quarterbacks

**DOI:** 10.3390/sports8020018

**Published:** 2020-02-06

**Authors:** Matthew D. Powless, Jesse A. Steinfeldt, Shelbi E. Fisher, Patrick McFadden, Kyle W. Kennedy, Scott Bellini

**Affiliations:** 1 Psychology Department, University of Southern Indiana, Evansville, IN 47712, USA; 2 Department of Counseling and Educational Psychology, Indiana University, Bloomington, IN 47405, USA; jesstein@indiana.edu (J.A.S.); shefishe@iu.edu (S.E.F.); pmcfadde@iu.edu (P.M.); kwkenned@imail.iu.edu (K.W.K.); sbellini@indiana.edu (S.B.)

**Keywords:** decision making, working memory, temporal occlusion

## Abstract

Despite the role of working memory capacity (WMC) in decision making, there is a dearth of empirical literature concerned with working memory and how it relates to tactical decision making in sport. The temporal occlusion paradigm has often been used by sport researchers to improve tactical decision making and, thus, provides a well-established foundation for creating decision-making trainings. Therefore, the purpose of the current study was to explore the implementation of computer-based learning modules to improve the tactical decision making of four high school quarterbacks with varying levels of WMC, utilizing a single-subject, multiple baseline design. The learning modules utilized a temporal occlusion paradigm and present a novel intervention aimed at improving decision making in quarterbacks. Data were analyzed using visual analysis and improvement rate difference (IRD). Overall, results did not demonstrate a causal relationship between changes in accuracy of decision making after implementation of the learning modules but did provide moderate evidence for improvement in reaction time for decision making due to the learning modules. The learning modules were met with positive perceptions from the four participants, and the participant with the lowest WMC showed evidence of improvement in both accuracy and speed of decision making. Limitations as well as implications will be discussed.

## 1. Introduction

In team-based sports, athletes must often attend to many stimuli and engage in open-skill tasks where many response options are available [1]. This may include playmakers such as a point guard in basketball who may need to handle the ball, call a play, read a defense, and then decide whether to pass to a teammate, retain possession of the ball, or pull up and shoot. It may also apply to a quarterback in United States (U.S.) football, who must read a defense, recognize the best available options based on the defensive scheme, his personnel, and the intended play, call an audible if necessary, and then once the ball is snapped, choose whether to pass to a teammate or keep the ball and rush for yardage. These athletes have the difficult and complex task of attending to many stimuli in a short period of time in order to make appropriate tactical decisions. While many variables impact this process, an integral component to their tactical decision-making ability is their working memory (WM; [2]).

WM includes tasks that require conscious processing and attention (e.g., selecting a receiver to throw to) rather than automatized tasks (e.g., throwing a football for a skilled quarterback). Yet despite its important role in tactical decision making, there is little research investigating WM in sport and scholars have commented that WM would be a beneficial avenue for sport researchers to study (for further review, see [2,3]). Indeed, empirical research investigating the role of WM in sport has begun to emerge (e.g., [4]). Furley and Memmert [4] conducted experimental studies investigating the role of working memory capacity (WMC) in the decision-making processes of basketball and ice hockey players. In computer-based, sport decision-making tasks, they found athletes with high WMC to be better able to ignore irrelevant auditory distractions and focus their attention on making tactical decisions and also found athletes high WMC to be better able to adapt their tactical decision making depending on what situations were presented to them, rather than relying on the prepotent instructions of a virtual coach [4].

In addition to burgeoning studies, scholars have provided many recommendations for how to investigate WM and/or decision making with athletes [2,4,5,6]. Such recommendations for research in WM and/or decision-making tasks in sport have included, but are not limited to: exploring WM as a potentially moderating variable in sporting behavior (e.g., decision making; [2]), using sport-specific trainings to improve the tactical decision making of athletes rather than traditional computer-based WM trainings [4], and using segmented perceptual video trainings to improve decision making—specifically, trainings that call on the same cognitive processing scheme that would be utilized in the actual sport scenario [5]. The present study aims to integrate scholarship from the areas of WM and perceptual trainings in order to create and evaluate a novel tactical decision-making intervention for high school quarterbacks in U.S. football. To set the stage for this intervention, it is important to first more thoroughly review WM and its role in sport.

### 1.1. Working Memory

WM is an interesting area of inquiry in sport research because WM is responsible for maintaining, updating, and retrieving information that is relevant to an individual’s task goal while suppressing or ignoring irrelevant information—key skills to success in sport [4,7]. Past models of WMC (e.g., [8]) refer to WMC as the ability to hold a certain amount of information available in WM, but more recently, the controlled attention theory of WM defined WMC as “the attentional processes that allow for goal-directed behavior by maintaining relevant information in an active, easily accessible state outside of conscious focus, or to retrieve that information from inactive memory, under conditions of interference, distraction, or conflict,” [9] (p. 23). Thus, contemporary models of WMC have focused on how attention is allocated when processing information during WM tasks, rather than the amount of information that can be stored in WM [4,10].

The controlled attention theory of WMC aligns with two key models of working memory: Baddeley’s [11,12] central executive and Norman and Shallice’s [13] supervisory attentional system (SAS). These two models were formed to explain how attention is allocated during WM tasks. The SAS (which was adopted by Baddeley as a framework for the central executive, [12]) can be used to depict a helpful example for understanding how attention is delegated when completing tasks. Under normal circumstances, an individual driving their car to work may be a fairly automatic task that will not require much conscious attention. On the other hand, if it is a particularly rainy day and there is construction on the way to work, the SAS may override that individual’s normal behavior patterns so that they can direct their attention to consciously finding a new route to work. Likewise, in sport, certain tasks may be automatic for athletes (e.g., throwing a football to a receiver), but when presented with interfering stimuli (e.g., defenders rushing the quarterback), WM may have to override automatic tasks and allocate attention for how to successfully complete the task in the face of interference (e.g., evading defenders before throwing to a receiver). Theoretically, those with higher WMC should be more capable of appropriately allocating attention so that they can effectively cope with interfering stimuli in order to select appropriate decisions in sport situations. This theoretical assumption has gained some empirical support in sport research [4].

Furley and Memmert (Experiment 2; [4]) found low WMC on traditional WM tasks to be associated with athletes blindly following inappropriate, prepotent instructions during a tactical decision-making task. Although the authors found a relationship between WMC and tactical decision making, it is still unclear whether high WMC is necessary to make appropriate tactical decisions during sport performance [14]. Trainings aimed at improving WMC appear to only improve WMC in the area for which participants are trained [15]. Given these caveats, Furley and Memmert [4] suggested that coaches would be better advised to incorporate sport-specific training to improve the tactical decisions of their athletes rather than incorporating traditional, computer-based WM training sessions. This recommendation is in alignment with working memory models (e.g., long-term working memory; [16]), which propose that experts are able to make effective decisions because of their extensive knowledge base and past experience with many situations in their field of interest. One effective tool for expanding the knowledge base of athletes and improving their decision-making ability has been temporal occlusion interventions.

### 1.2. Temporal Occlusion Interventions in Sport

Decision-making trainings in sport are often conducted through the progressive temporal occlusion paradigm. The progressive temporal occlusion paradigm typically involves taking dynamic visual images, often from the athlete’s perspective, and editing them so that they occlude over different time periods during the action of an opposing player (for further reading, see Farrow, Abernethy, and Jackson, 2005 [17]). This paradigm is useful in testing athletes’ ability to rapidly use sport-relevant cues to make accurate predictions and decisions (i.e., advanced cue utilization; [5]).

Expert athletes are generally better decision makers in their domain of expertise than their novice peers, due to their visual search behavior, use of relevant cues, and extensive knowledge base [5,18,19,20]. With enough practice though, temporal occlusion interventions have been effective in improving the prediction/decision-making accuracy of intermediate-level athletes [21], high-level adolescent athletes [22], and elite-level athletes [23]. Most studies using the temporal occlusion paradigm investigate processes involved in interceptive activities, such as predicting the direction of a penalty kick in soccer [23,24,25], fielding and batting in cricket [26,27], returning a tennis serve [21], catching a ball one-handed [28], predicting the landing position of a shuttle in badminton [29], and predicting the direction and force of an opponent’s stroke in squash [30].

To this author’s knowledge, no study has utilized the temporal occlusion paradigm as an intervention to improve decision making in team-based sports where athletes are engaging in open-skill tasks for which they must attend to many competing stimuli. The closest example of such a study (to this author’s knowledge) was conducted by Ward, Ericsson, and Williams [31] with soccer players. In their study, skilled and less-skilled soccer players were presented with occluded video from soccer games [31]. In one occlusion paradigm (Experiment 2; [31]), the authors occluded video of a soccer player about to make a tactical decision at least 120 ms prior to the player’s foot–ball contact (only one occlusion rather than a progressive occlusion). Participants had to first predict what action their opponent would engage in (e.g., pass, shoot, retain possession) as well as what direction they would go in. Participants were then asked to generate a list of options that were available to their opponent (e.g., a shot on the goal, retaining possession of the ball, a pass) and then rank order the threat each option posed to their team. The authors found that skilled players were better able to predict the action of their opponent than less-skilled players. Additionally, skilled and less-skilled players both generated only a few options for their opponent (M = 2.02, SD = 0.43) [31], but skilled players generated more task-relevant options and less task-irrelevant options. The data demonstrated that experts are able to make accurate predictions and situational assessments even in the absence of retrieval cues (i.e., video images) at the time of reporting [31].

### 1.3. Current Study

Building on the work of Furley and Memmert [4] and Ward and colleagues [31], the temporal occlusion paradigm may be a valuable way to improve the decision-making processes of athletes engaging in open-skill tasks in team-based sports. This type of intervention forces the participating athlete to attend to cues rapidly and form appropriate responses quickly.

As such, the current study aimed to integrate the lines of research in WM and temporal occlusion interventions to create a novel tactical decision-making intervention for athletes in a team-based sport. Specifically, this intervention was intended for high school quarterbacks in U.S. football. Therefore, using a modified temporal occlusion paradigm as a training intervention, we aimed to answer the following four questions:

1. How might a temporal occlusion intervention impact high school quarterbacks’ ability to recognize defensive schemes?
2. How might a temporal occlusion intervention impact the pass selection of high school quarterbacks?
3. How might a temporal occlusion intervention impact the ability of high school quarterbacks, with varying levels of WMC, to adapt their tactical decisions appropriately when faced with new stimuli?
4. What are high school quarterbacks’ perceptions of a perceptual training utilizing a modified temporal occlusion paradigm?

Based on literature demonstrating the positive training effects of interventions utilizing a temporal occlusion paradigm, e.g., [21,22,23], suggestions to use sport-specific trainings to improve decision making rather than traditional WM trainings [4], and athletes’ tendency to want to engage with temporal occlusion interventions when given the choice [23], our hypotheses were:

1. High school quarterbacks’ ability to recognize defensive schemes will improve after completing a temporal occlusion intervention.
2. High school quarterbacks’ pass selection will improve after completing a temporal occlusion intervention.
3. High school quarterbacks will improve in their ability to adapt their decisions appropriately, regardless of their WMC, following the completion of a temporal occlusion intervention.
4. High school quarterbacks will have positive perceptions of a perceptual training utilizing a modified temporal occlusion paradigm.

## 2. Materials and Methods

### 2.1. Participants

Participants for the current study were four male student-athletes from the Midwest U.S. with ages ranging from 14 to 18 years old—all of whom played the position of quarterback in football. All participants went to the same high school and participated in the same high school football program, with the exception of one student, who would be attending the high school and competing in the football program the academic year following data collection. The primary inclusion criterion was that the participants had to play quarterback for their respective football team within the program (e.g., varsity, junior varsity, etc.). Below is a brief description of each participant. Pseudonyms have been used to protect their confidentiality.

#### 2.1.1. Aaron

Aaron was a 15-year-old, White high school sophomore at the time of this study. He had played football for nine years and the position of quarterback for six years. In the season prior to this study, he had split time between junior varsity and varsity for the high school’s football team and described himself as a non-starter on the varsity football team. He estimated that he watches approximately 3–4 h of film (i.e., video of football games) each week in general (inclusive of NFL games, his high school team’s games, etc.). He estimated that he watches 3–4 h of his high school team’s film in-season and 0–1 h of his high school team’s film out-of-season. In addition to playing football, Aaron played golf.

#### 2.1.2. Brett

Brett was a 17-year-old, White high school junior. Brett had played football for the past six years and had played the position of quarterback for five of those six years. The past season, he was a starter on the varsity football team and played baseball as well. Brett reported that he watched approximately 3–4 h of the team’s film each week in-season and approximately one hour each week in the off-season. He reported watching 12–20 h total of football film each week.

#### 2.1.3. Cam

Cam was a 14-year-old, multi-racial eighth-grade student. Cam had played football for eight years and the position of quarterback for seven years. During the football season prior to this study, he was the starting quarterback for his eighth-grade team. Cam estimated that he watches film for one hour a week in general, one hour a week of his team’s film when his team is in-season, and zero hours per week of his team’s film when his team is out-of-season. Cam was a multi-sport athlete who played basketball, baseball, and track-and-field in addition to football.

#### 2.1.4. Dan

Dan was a 15-year-old, Black freshman in high school. He reported playing football for the past five years and quarterback for two years. In the prior season, he played at the freshman level for his high school’s football program and indicated that he was a starter for the freshman team. Dan reported watching on average 4–5 h of football film in general each week, four hours of his high school team’s film each week in-season, and 1–2 h of his high school team’s film out-of-season. He also indicated that he played basketball for his high school as well. Demographic information and individual characteristics are summarized in Table 1.

### 2.2. Measures

#### 2.2.1. Counting Span Task

The counting span task [10,32,33,34] is a traditional WM task that was used to measure the domain general WMC of the participants. The purpose of having a WMC measure was to add to past research [4] as well as observe whether WMC was related to the outcomes that participants attained on the dependent variables of interest (i.e., accuracy and reaction time). The reason the counting span task was chosen was because the simplicity of the processing component of the task (i.e., counting) makes it ideal for many populations [35].

In the counting span task, participants are presented with a gray display that contains 3–9 dark blue circles, 1, 3, 5, 7, or 9 dark blue squares, and 1–5 light green circles. The number of dark blue circles, dark blue squares, and light green circles is approximately balanced across tasks [10]. Generally, during the task, participants count aloud the number of dark blue circles on a screen, and once they are done with their count, they state how many dark blue circles they counted (this is considered one item) and the researcher presses a key on the computer to move to the next stimulus display [4]. In the current study, participants moved to the next screen by pressing the appropriate keys on their own. After 2–6 stimulus displays, participants are presented with a recall cue, a screen with question marks presented on it, which prompts participants to record the number of dark blue circles they saw on each screen in the correct order in which they saw them. The amount of stimulus displays between one recall cue and the next is considered one set. Therefore, each set may have 2–6 stimulus displays—each with one correct item (i.e., the number of dark blue circles) [4]. Participants completed 15 sets in total [35].

#### 2.2.2. Stimuli

For the current study, in order to create a decision-making test and learning modules for the participants, clips of passing plays from the high school’s previous season were downloaded from the team’s hudl account [36]. By downloading clips from the team’s hudl account, fidelity to the sporting environment the athletes compete in was increased [5], because athletes were able to watch their team, running their offense, utilizing the cognitive scheme their coach teaches, against teams they have played, and in many cases, teams they will continue to play. Additionally, for two participants, Aaron and Brett, they would be likely to see themselves running the offense in the clips used, as Aaron or Brett was playing quarterback in the majority of clips used. Having some participants learn from themselves provided a form of self-observation, which has been found to be effective in improving self-efficacy and performance for a variety of athletes [37]. Clips were filmed from a sideline view and were edited using iMovie 10.1.4 (Apple Inc., Cupertino, CA, USA) to create stimuli to be used in a decision-making test as well as in learning modules, which are described below.

Only passing plays where all 11 defenders could be seen in the film were included as possible candidates for the study, leaving 119 plays as possible stimuli. Prior to video editing, the first author met with the quarterback coach of the football team to discuss the study and its purpose. The quarterback coach had 12 years of experience as a high school football coach. During the meeting, he described the cognitive process he instructs quarterbacks to go through when making tactical decisions, which were: identify possible coverages prior to the snap of the football from the center (i.e., the moment before the center snaps the ball), identify the coverage “at the snap,” (i.e., when cornerbacks have begun retreating 2–3 steps), and select a pass “post-snap,” (i.e., once the quarterback has the ball in hand and is looking downfield). Also of interest was the players’ ability to adjust their pass decision once the ball had been snapped if there were new, interfering stimuli (e.g., blitzing linebackers). Therefore, based on this description, each play was edited into four different clips. A breakdown of how each play was edited into four different clips (i.e., one play = four clips) titled Time 1–Time 4 (T1–T4) is listed below:

- Time 1 (T1)—Clip began when teams broke from their huddles and ended the moment before the center began to snap the ball to the quarterback.
- Time 2 (T2)—Clip began at the moment of the snap and ended when defenders (i.e., cornerbacks) had taken their initial 2–3 steps.
- Time 3 (T3)—Clip began at the moment of the snap and ended when the quarterback had the football in hand and was looking downfield. Often, T2 and T3 clips began and ended at the same points in time.
- Time 4 (T4)—Clip began at the snap and ended the moment before the quarterback released the football.

After the initial meeting, the quarterback coach went through the 119 plays identified as possible candidates and indicated whether he thought they were suitable to be used in the study (e.g., assessing if clips were too short, etc.). Additionally, if he thought the plays were suitable, he provided his answers to four questions to be asked in the intervention that coincided with the four time points outlined above. These questions would be asked after clips had occluded:

- T1—What coverage could this be?
- T2—What coverage is this?
- T3—What route should you play? (Questions at Time 3 and Time 4 are referencing what pass the quarterback should throw based on the play-call and routes the receivers are running.)
- T4—Which option would be best now?

Once the quarterback coach concluded reviewing the plays, there were 107 plays that he thought were suitable for the study. These 107 plays were reviewed by two more football experts. One expert had 22 years of experience as a high school football coach (18 years as a head coach), five years as a college coach, and multiple coaching awards. The other expert had 10 years of experience as a college football coach, seven years of experience as a high school coach (six years as a head coach), and played football at the collegiate level as well as professionally in Europe. Each expert was emailed a packet of worksheets and the edited clips for each play. On the worksheet, the questions that coincide with each clip were provided, and the coaches indicated their answers from a list of multiple choice options. After the experts completed and returned their worksheets, their answers were reviewed along with the quarterback coach’s answers. Any play where the same two experts agreed on each of the four decisions that had to be made at each point in time was included as a possible play for the study. The reason for choosing the same two experts agreeing on each of the four decisions is because this suggested that the experts had consensus on the decisions to be made throughout the cognitive progression of each play. This left 51 plays (212 clips) left to be included in the decision-making test and learning modules.

Once the final set of 51 plays had been selected and the 212 clips for each play created, the lead author consulted with one of the external experts on the content validity of the clips. The experts’ primary recommendation upon review of the clips was allowing for more time to elapse at T2, as some clips were too short to observe what was occurring if the film was occluded. This lead to the lengthening of some T2 clips. Clips at T2 were supposed to occlude after the cornerbacks’ first 2–3 steps had been taken. However, during the video editing process, some clips had ended short of this criteria and a cornerback had only taken a single step or no steps before the video was occluded. Additionally, an adolescent high school football player (who provided their assent as well as had parental consent) piloted the decision-making test (to be described in the next section) and reported that the clips allowed enough time to pick up visual cues and make tactical decisions.

#### 2.2.3. Decision-Making Test and Learning Modules

The first dependent variable of interest was the participants’ accuracy on questions T1–T4 on a decision-making test (DMT), which was collected using E-Prime 3.0 (Psychology Software Tools, Sharpsburg, PA, USA) [38]. A second dependent variable of interest was the participants’ reaction time (RT) on the DMT items. RT was also recorded using the E-Prime 3.0 software. RT was of interest because according to multiple decision-making theories (e.g., long-term working memory, recognition-primed decision, take-the-first) [16,39,40], as athletes gain experience, their first response selection to a tactical question will be more likely to be an appropriate response. Therefore, it was suspected that as expertise increased, participants would improve their accuracy in making “correct” decisions as well as make appropriate responses more quickly. The DMT consisted of 11 plays (one practice play and 10 test plays) created from a subset of the final 51 plays that remained after vetting. Additionally, the intervention, a set of five learning modules (LMs), with eight plays within each module was created from this same sub-set 51 plays.

In the DMT, participants were presented plays in E-Prime 3.0 that were edited as described previously. Prior to each play, participants were informed what the play call was, then hit the space bar on their keyboard when they were ready to begin. After the play-call, participants viewed a cross-hair centered on their screen for one second (s) and then watched one of the clips presented to the coaches. Clips progressed sequentially from T1–T4 for each play as it had for the coaches. However, the coaches had the advantage of rewinding, pausing, or watching plays without them being occluded (similar to [31]). Once the participants reached the end of the clips, the clips occluded, and the participants were presented a slide that asked the questions that coincided with each clip. Clips ranged from 0.3 to 5.0 s in length.

The DMT was created based off the proportion of defensive coverage schemes represented in the final 51 plays left in the stimuli after vetting. Out of the 51 plays, there were nine Man coverage plays, five Cover 1, five Cover 2, 20 Cover 3, 10 Cover 4, and two Cover 6 plays. Additionally, eight of the 51 plays involved the quarterback changing their decision at T4 from their previous answer at T3 due to new visual information, based on coach responses. The DMT consisted of one practice play (i.e., four practice clips) to orient the participant to the task and 10 test plays (i.e., 40 test clips). In the DMT, there were three Cover 3, two Cover 4, two Man coverage, one Cover 1, one Cover 2, and one Cover 6 plays. Additionally, there were two plays where the quarterbacks needed to change their pass selection at T4 from their original pass selection at T3. Similar to Furley and Memmert [4], when changing a decision at T4 from the one given at T3, the pair of decisions was titled an “adaptation set.” Participants’ accuracy on adaptation sets was a tertiary dependent variable of interest. The low ratio of sets for when the quarterback had to change their decision at T4 in the DMT was chosen because it was believed that WMC differences would be more pronounced if the athletes could consistently rely on automatic responses [4].

After answering questions at T4, participants were asked a fifth question, which asked what pass they selected at T3. The rationale for asking which pass was selected at T3 was that individuals are more likely to miss visual cues if they have to remember and recall targets at a later point in time [4,41]. Per the recommendations of Kratochwill and colleagues [41] there were four different versions of the DMT. Each version contained the same plays, but plays were randomly assorted within each DMT so that participants were never presented plays in the same order two times in a row when the DV was collected.

The independent variable for this study was a set of five LMs. The LMs were similar to the DMT with a few exceptions. In LMs, participants answered questions for eight plays (i.e., 32 clips) rather than 10 plays as in the DMT. After participants answered the questions at T1–T4, they were provided feedback regarding the accuracy of their answers. If their answer was incorrect, they were shown the clip again and asked to answer the coinciding question again. If participants answered a clip’s question correctly, they would proceed to a slide that provided evaluative information explaining why their answer was correct from an expert (i.e., the quarterback coach). This evaluative information was a form of feedforward [42], directing participants to relevant visual cues within clips to help inform their visual search process when watching clips in the future. Feedforward information also helped control for circumstances when a player “guessed” correctly, so that participants were still able to learn whenever they answered a question correctly, even though they may not have known “why” they were correct. Within this slide, participants could also click a radio button on the bottom of the slide that allowed them to re-watch the clip as many times as they would like or press the space bar on their keyboard to move forward to the next clip. Lastly, after answering the fourth question at T4 correctly, participants were again asked a fifth question, which asked them what answer was correct at T3. Again, the purpose of this fifth question was to task participants with having to remember and recall a target at a later point in time, as they did in the DMT.

It is worth noting that only one play was used twice in the LMs because it was a Cover 6 play. Since there were only two plays illustrating Cover 6 in the pool of possible plays to be included, and one was used in the DMT, it was decided to include this play twice so that participants had more exposure to it. The remaining 38 plays used in the LMs were only shown once each, allowing for a great deal of uniqueness to each LM.

#### 2.2.4. Social Validity Scale

After the third LM and fifth LM, participants completed social validity measures to assess the practicality of the intervention [43]. Surveys contained seven statements that participants could indicate their agreement with on a 4-point Likert scale ranging from “strongly disagree” to “strongly agree.” The survey questions addressed concerns related to (a) perceived effectiveness of the intervention, (b) interference with sport activities, (c) extent to which participants found the LMs enjoyable, (d) practicality of the intervention, and (e) importance of the tasks trying to be improved (i.e., accuracy and speed of decision making). Participants were also provided extra space to provide any additional comments they had regarding the intervention.

### 2.3. Procedure

The study was conducted according to the Declaration of Helsinki [44] and was approved by the university Institutional Review Board (IRB). The first author obtained parental consent and participant assent prior to the start of the study. Once parental consent and participant assent had been received, participants completed the demographic questionnaire and were individually administered the Counting Span Task [10,32,33,34] prior to the beginning of the baseline phase.

#### Design

The current study utilized a single-subject, multiple baseline design. Prior to collecting data, it was determined that each participant would complete the DMT at least three times before moving into the intervention phase in order to meet criteria set forth by Kratochwill and colleagues [45]. During the baseline phase, Aaron, Brett, and Dan met with the first author during their school’s study hall period twice a week to complete the DMT and establish baseline data. All three participants met in a group setting, each participant completing their respective DMTs on separate laptops in the same room. Participants were instructed to not discuss their answers to the DMTs. During the baseline phase, participants completed two alternative forms of the DMT at each meeting. Participants completed one DMT, took approximately a five-minute break, and then took another form of the DMT. Participants remained in the testing room during their break so that the researcher could confirm that they did not discuss answers. Additionally, they were not provided feedback on how they did on the DMT (with regards to accuracy or reaction time of answers). Cam completed the DMTs individually after school. He followed the same procedure during baseline phase as the other participants. Participants never took the same form of the DMT twice in the same day. A fidelity checklist was completed at each meeting. The checklist indicated which DMTs each participant was to take, and the researcher used it to confirm that the participants were administered the appropriate DMT at each meeting. The session schedule for participants at baseline and intervention phase can be found in Table 2.

Once baseline data had been established for a participant, they were introduced into the intervention phase. At each meeting during the intervention phase, participants completed one LM, took a five-minute break, then completed a DMT. Similar to the baseline phase, in order to maintain fidelity to the intervention [45], there was an intervention fidelity checklist that indicated what LM a participant should take each session, what DMT they should take, and if they should complete the social validity scale. In order to stay true to a multiple baseline design, participants were introduced to the intervention in a staggered format such that no participants started the intervention on the same day. This allowed for eight unique phases and four phase shifts over the course of the intervention, optimizing experimental control.

### 2.4. Data Analysis

Prior to administering DMTs, participants’ WMC as measured by the Counting Span Task was calculated. Having a way of examining the athletes’ domain general WMC was helpful to have a more comprehensive understanding of the intervention’s impact on participants. WMC was found by using a partial credit load score on the Counting Span Task [10,32,33,34]. A partial credit load score [35] meant that a correctly recalled item in a set of two items was worth 2 points, a correctly recalled item in a set with three items was worth 3 points, etc. Points were then summed and divided by the total score possible for the set [4].

The primary method of data analysis for this study was visual data analysis, which inspects level, trend, variability, immediacy of the effect, overlap, and consistency of dependent variable data patterns across similar phases in order to assess for within- and between-phase patterns [45]. Data analysis methods followed the best practice guidelines for single case research outlined by the What Works Clearinghouse Procedures and Standards Handbook [45], and included an analysis of the immediacy of change in the dependent variable following the introduction of the independent variables, the magnitude of change from baseline to intervention, the variability in responding across phases, and an analysis of the slope and direction of the dependent variables across phases. The effectiveness of the intervention was determined by both visual inspection of the graphical representation of the data, and statistical analysis of the dependent variable. A trend analysis was conducted via the analysis of the slope of the dependent variables for both the baseline and intervention phases. The magnitude of change was determined by visual inspection of the graphs and the change in mean performance for each phase of the study. In order to create graphs that could be visually inspected, participants’ data were transferred from E-Prime 3.0 [38] to Microsoft Excel 14.6.3. This transfer to Excel allowed the establishment of inter-assessor agreement, meeting evidence standards [45]. The dependent variables under inspection included: percentage of correct answers on DMTs (in recognizing defensive schemes and selecting passes), mean RT when responding to items on DMTs (both defensive recognition and pass selection items), and accuracy of responses on adaptation sets.

Once a participant’s pattern of responding on the DMT had become consistent in the baseline phase (with regards to accuracy of answers), the independent variable was introduced. Consistency in the baseline phase was achieved when a participant had demonstrated a documented, predictable pattern of responding on the DMT that was observed via visual data analysis as recommended by Kratochwill and colleagues [45]. No two participants were introduced to the intervention phase on the same day to allow for four unique phase shifts and to optimize experimental control. In order to analyze within phase patterns, level (mean score for the data within a phase), trend (slope of the best-fitting line within a phase), and variability (range or standard deviation of data around the line of best-fit) were examined [45]. Once the intervention was introduced, to assess for between-phase patterns, the immediacy of the effect and overlap were examined [45]. Immediacy of the effect refers to the change in level between the last three data points in one phase and first three data points of the next phase. Overlap refers to the proportion of data from one phase that overlaps with data from the previous phase [45]. Overlap was utilized to determine effect sizes for the intervention.

Intervention effect size was calculated using improvement rate difference (IRD) [46,47]. This form of analysis can be conceptualized as the difference in improvement rates between Phase A and Phase B [46,47]. In order to calculate IRD, the minimum number of overlapping scores (scores on dependent variables in the intervention phase that are less than or equal to scores in the baseline phase) from the participants’ baseline and/or intervention phase are removed to eliminate all overlap between phases [46,47]. Once removed, a ratio of improvement would be constructed for each phase, which is the number of improved data points divided by the number of total data points in order to calculate improvement rate (IR; # improved/# total = IR). An improved data point in the baseline phase (Phase A) is any data point that is greater than or equal to any data point in the intervention phase, and an improved data point in the intervention phase (Phase B) is any data point that is greater than all data points at baseline. The difference between IR for Phase A and Phase B is then found in order to find IRD (IR_B_ − IR_A_ = IRD) [46,47]. The maximum score for IRD is 1.00 or 100%, with scores less than 50% having very small and questionable effects, scores between 50% and 70% having moderate effects, and scores greater than 70% having large effects [46]. IRD methods have improved statistical precision and less vulnerability to outliers compared to other overlap procedures, such as Percent Non-Overlapping Data (PND) and Percentage of Data Exceeding the Mean (PEM) [46]. Data were analyzed with regards to participants’ performance overall (responses to T1–T4 items), performance in recognizing defensive schemes (T1–T2 items), performance in selecting passes (T3–T4 items), and adaptation sets (two per DMT).

With regards to the current study, according to Kratochwill et al. [45], there may be strong evidence for a relationship between the independent variable and dependent variable if there was demonstration of an effect caused by the independent variable for all four participants. If an effect attributable to the independent variable was not demonstrated for at least three of the participants, then there would be no evidence of a causal relationship between the independent variable and the outcome variable. If an effect was demonstrated for three participants and there was no effect for a fourth participant, then there would be only moderate evidence for the relationship between the independent variable and outcome variable [45].

## 3. Results

**Figure 1:**
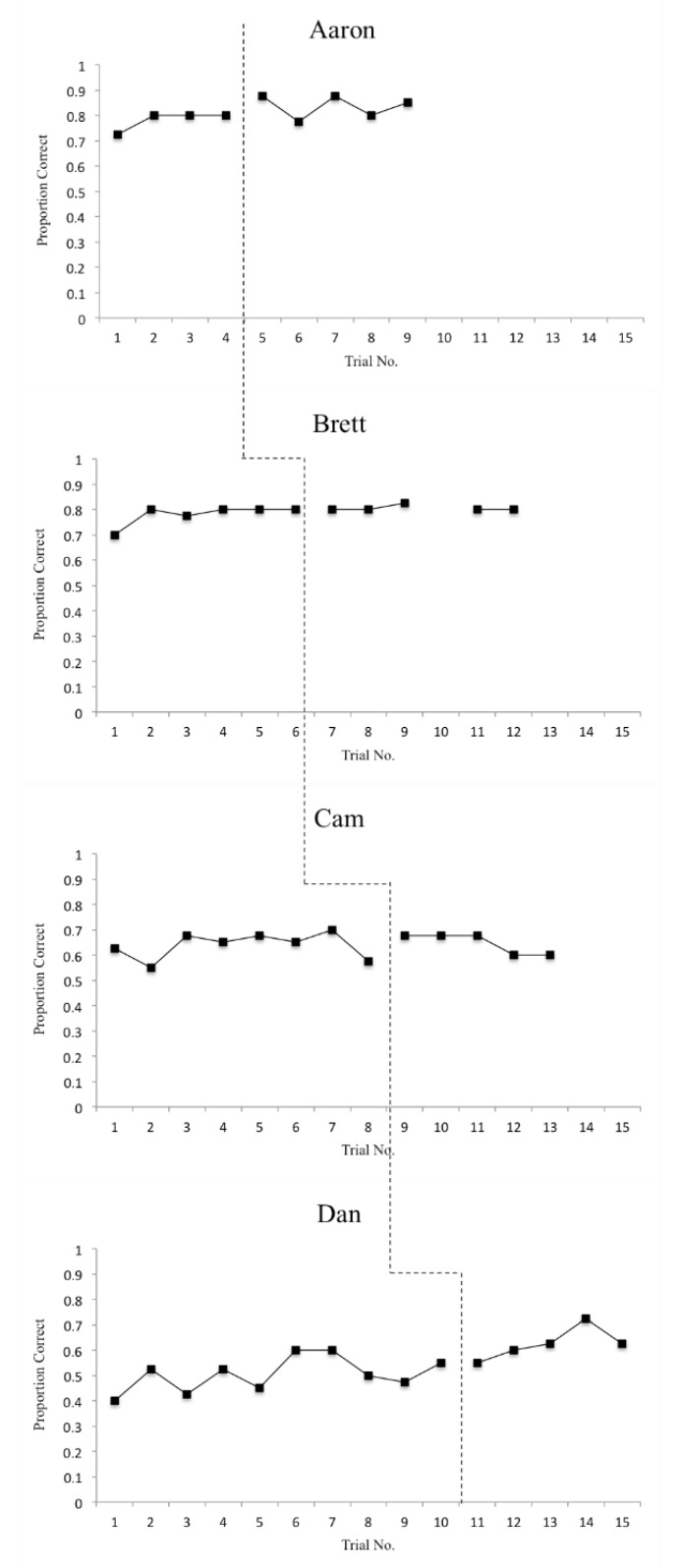
Proportion of correct answers of participants on decision-making tests (DMT)s overall.

**Figure 2:**
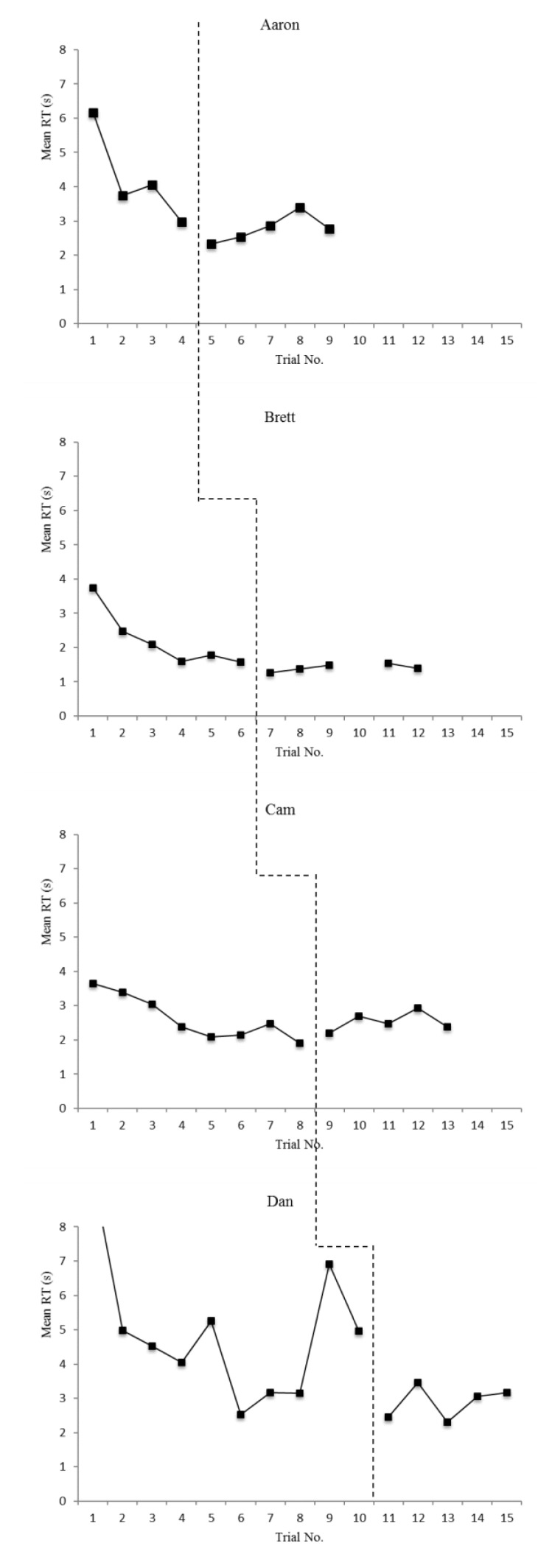
Mean reaction times (s) on DMTs overall (Dan’s initial baseline session mean reaction time was 9.51 s, which was an outlier data point that is not extends beyond the y-axis of the graphs above).

**Figure 3:**
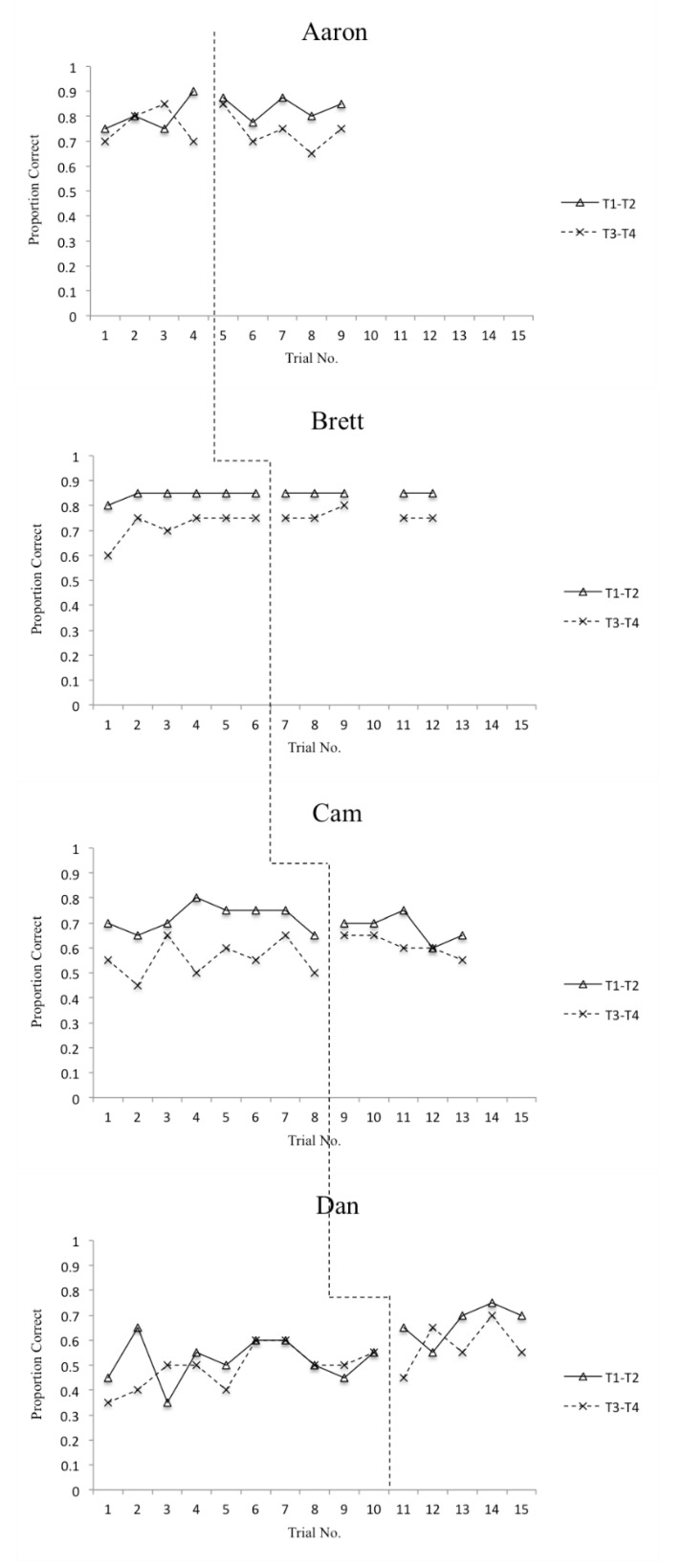
Proportion of correct answers on DMTs for T1–T2 and T3–T4 items.

**Figure 4:**
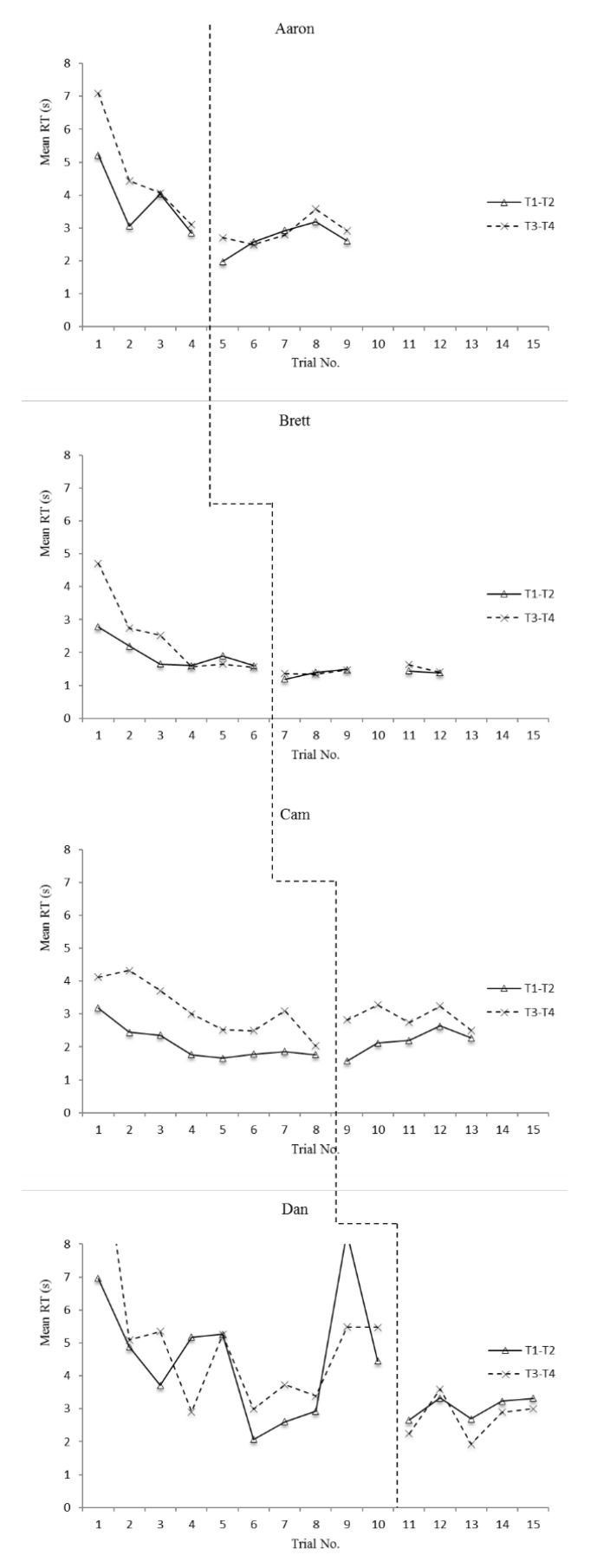
Mean reaction times (s) on T1–T2 and T3–T4 items (Dan had a mean reaction time of 8.34 s for T1–T2 in his 9th baseline measurement of the dependent variable. This was an outlier that extends beyond the y-axis in the graphs above).

**Figure 5:**
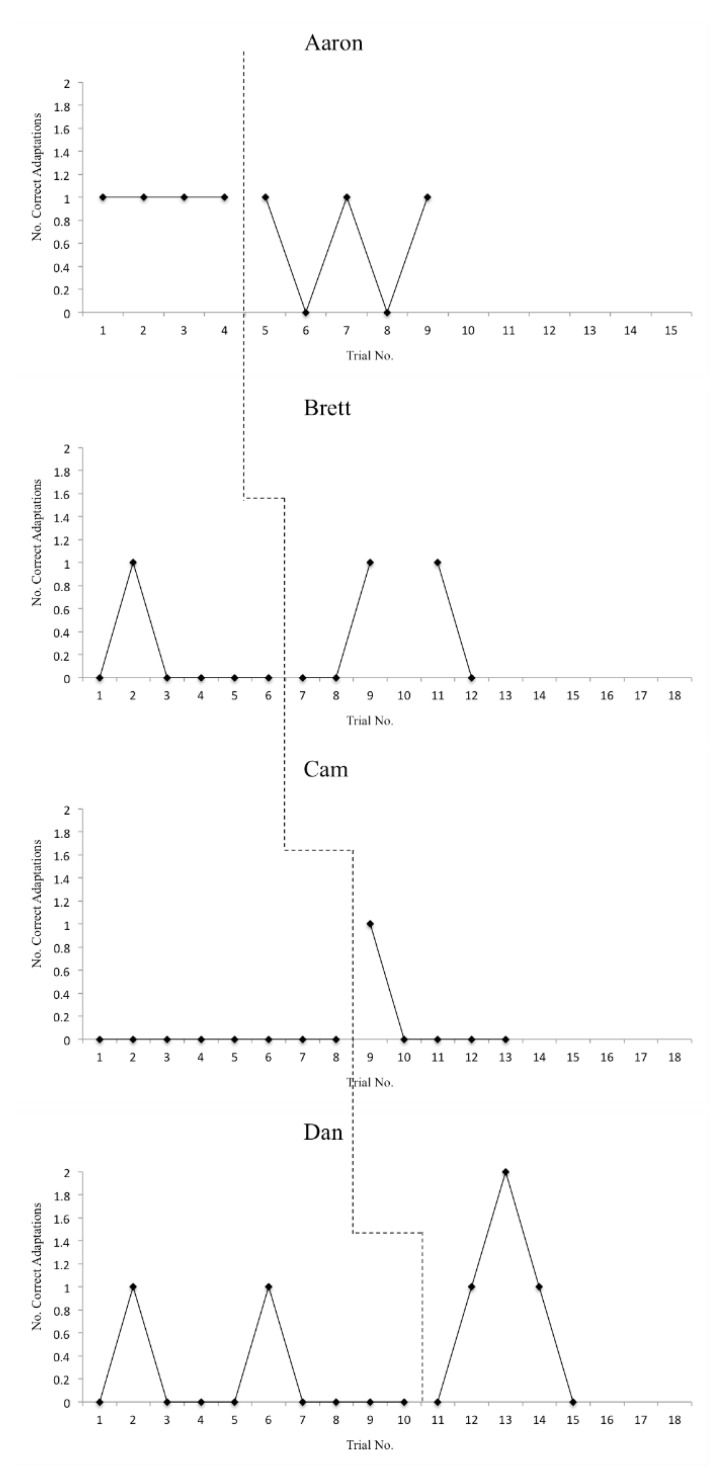
Number of correct adaptations for adaptation set items.

The means and standard deviations for accuracy and reaction time on T1–T2 and T3–T4 items for each participant are displayed in Table 3 and Table 4, respectively. Additionally, overall (T1–T4) accuracy scores are displayed graphically in Figure 1 and overall (T1–T4) reaction time scores are displayed graphically in Figure 2. In order to address the first two research questions, defense recognition (T1–T2) and pass selection (T3–T4) accuracy and reaction time data are displayed graphically in Figure 3 and Figure 4. The number of correct adaptations is displayed graphically in Figure 5. Lastly, IRDs for accuracy and reaction time for each participant can be found in Table 5. These tables and figures have been included in the current section following the participants’ reported results.

### 3.1. Aaron

#### 3.1.1. Overall (T1–T4)

Aaron had a WMC of 0.70, which was second highest among the participants. Aaron had a mean baseline accuracy of 78.13%, with a relatively flat trendline, ranging from 72.50% to 80% (SD = 3.75%). In the intervention phase, Aaron’s mean accuracy increased to 83.50% with a flat trendline, ranging from 77.50% to 87.50% (SD = 4.54%). Aaron’s baseline reaction time had a mean of 4.23 s (s), with a decreasing trendline, ranging from 6.16 s to 2.97 s (SD = 1.36 s). Aaron’s mean reaction time in the intervention phase was 2.78 s, with a slightly increasing trendline, ranging from 2.34 s to 3.39 s (SD = 0.40 s). IRD was 60% for accuracy and 80% for reaction time, indicating moderate improvement for accuracy and large improvement for reaction time across phases for Aaron.

#### 3.1.2. Defense Recognition (T1–T2)

With regards to recognizing defensive schemes, Aaron had a mean accuracy score of 80% (SD = 0.07) with an ascending trendline. Aaron’s mean accuracy score in the intervention phase was 93% with a relatively flat trendline. Aaron’s mean reaction time at baseline was 3.79 s (SD = 1.08 s), with a decreasing trendline. Aaron’s mean reaction time in the intervention phase was 2.66 s (SD = 0.46 s). IRD between baseline and intervention phases 60% and 55%, respectively, suggesting a moderate effect for both.

#### 3.1.3. Pass Selection (T3–T4)

Aaron’s baseline accuracy on pass selection items had a mean of 76.25%, with variable data (SD = 2.24%) and a flat trendline. Aaron’s mean accuracy score during the intervention phase was 74% with a descending trendline (SD = 7.50%). Aaron’s baseline reaction time had a mean of 4.67 s (SD = 1.71 s) with a decreasing trendline. The mean reaction time during the intervention phase was 2.90 s (SD = 0.41 s), and there was a slightly ascending trendline. IRD was −10% for accuracy data, indicating no improvement, and was 80% for reaction time data, indicating strong improvement. Lastly, with regards to the adaptation sets on the DMT, Aaron correctly answered four adaptation sets out of eight in the baseline phase (50%) and one out of 10 (10%) in the intervention phase. Between baseline and intervention phases, Aaron had an IRD of −60% on adaptation sets, indicating no improvement.

### 3.2. Brett

#### 3.2.1. Overall (T1–T4)

Brett had the highest WMC score with a score of 0.86. Brett’s baseline accuracy had a mean of 77.92% with a flat trendline, ranging from 70% to 80% (SD = 4.00%). Brett had a mean reaction time of 2.21 s at baseline, with a decreasing trendline, ranging from 1.58 to 3.74 s (SD = 0.83 s). Brett’s mean accuracy increased to 80.50% in the intervention phase with a flat, very consistent trendline that ranged from 80% to 82.50% (SD = 1.11%). Brett’s mean reaction time decreased to 1.41 s during the intervention phase, with a flat trendline, ranging from 1.27 to 1.53 s (SD = 0.10 s). Brett had an IRD of 33.33% for his accuracy scores, suggesting a very small/questionable improvement between baseline and intervention phase. However, Brett had an IRD of 100% for reaction time in the intervention phase, indicating a large improvement between baseline and intervention phase.

#### 3.2.2. Defense Recognition (T1–T2)

With regards to recognizing defensive schemes, Brett had a mean accuracy score of 84.17% (SD = 2.04%) with consistent data and a flat trendline. Brett’s mean accuracy score in the intervention phase was 85% (SD = 0%), again with consistent data and a flat trendline. Brett’s mean reaction time at baseline was 1.96 s (SD =0.47 s) with a decreasing trendline. Brett’s mean reaction time in the intervention phase was 1.38 s (SD = 0.11 s). IRD between baseline and intervention phases was 16.67% for accuracy and 100% for reaction time, indicating questionable improvement for accuracy and strong improvement for reaction time.

#### 3.2.3. Pass Selection (T3–T4)

For pass selection questions, Brett had a mean accuracy score of 71.67% (SD = 6.06%) at baseline with an ascending trendline. In the intervention phase, Brett’s mean went up to 76% (SD = 2.24%), and he had a flat trendline. Brett’s mean reaction time for pass selection items was 2.46 s (SD = 1.22 s) at baseline with a decreasing trendline. Brett’s mean reaction time in the intervention phase was 1.44 s (SD = 0.11 s) with a flat trendline. IRD for accuracy on pass selection items was 20% indicating questionable improvement and IRD for reaction time was 80%, indicating large improvement. Lastly, Brett correctly answered 1 adaptation set out of 12 in the baseline phase (8.33%) and 2 out of 10 in the intervention phase (20%). IRD between baseline and intervention phases on adaptation sets was 23.33%, indicating questionable improvement.

### 3.3. Cam

#### 3.3.1. Overall (T1–T4)

Cam had the third highest WMC with a partial-credit load score of 0.60. At baseline, Cam had a mean accuracy of 63.75% with a relatively flat, slightly ascending trendline. Accuracy data at baseline were fairly consistent, ranging from 55% to 70% (SD = 5.18%). Cam had a mean reaction time of 2.63 s at baseline with a decreasing trendline that was also fairly consistent, ranging from 1.90 to 3.65 s (SD = 0.65 s). During intervention, Cam had a mean accuracy of 64.50% with a consistent, decreasing trendline that ranged from 60% to 67.50% (SD = 4.11%). Cam’s mean reaction time during the intervention phase was 2.54 s with a consistent, flat trendline ranging from 2.20 to 2.94 s (SD = 0.29 s). Although Cam’s means improved across phases, this appears to have been due to low accuracy scores and high reaction times in the baseline phase. His IRD for accuracy was 22.50% and for reaction time was 37.50%, indicating questionable improvement in accuracy and reaction time across phases.

#### 3.3.2. Defense Recognition (T1–T2)

In recognizing defenses, Cam had a mean accuracy score of 71.88%% (SD = 5.30%) with slightly variable data and a flat trendline. Cam’s mean accuracy score in the intervention phase was 68% (SD = 5.70%) with a decreasing trendline. Cam’s mean reaction time at baseline was 2.10 s (SD =0.53 s) with a decreasing trendline. Cam’s mean reaction time in the intervention phase was 2.16 s (SD = 0.38 s) with an ascending trendline. IRD between baseline and intervention phases was −30% for accuracy and 20% for reaction time, indicating no improvement for accuracy and questionable improvement for reaction time.

#### 3.3.3. Pass Selection (T3–T4)

In selecting appropriate passes, Cam had a mean accuracy score of 55.63% (SD = 7.29%) at baseline with a slightly ascending trendline. In the intervention phase, Cam’s mean increased to up to 61% (SD = 4.18%), but he had a decreasing trendline. Cam’s mean reaction time was 3.16 s (SD = 0.82 s) at baseline with a decreasing trendline. Cam’s mean reaction time in the intervention phase decreased to 2.92 s (SD = 0.33 s) with a flat trendline. IRD was 15% and 37.50% for accuracy and reaction time, respectively, suggesting questionable improvement for both. Cam correctly answered zero out of 16 adaptation sets at baseline (0%) and one out of 10 in intervention (10%). IRD for adaptation sets was 35%, indicating questionable improvement.

### 3.4. Dan

#### 3.4.1. Overall (T1–T4)

Dan had the lowest WMC score with a score of 0.29. Dan’s baseline accuracy had a mean of 50.50% with a variable, increasing trendline that ranged from 40% to 60% (SD = 6.85%). Similarly, with regards to reaction time, Dan had a variable, decreasing trendline at baseline that ranged from 2.53 s to 9.51 s with a mean of 4.91 s (SD = 2.06 s). In the intervention phase, Dan had a mean accuracy score of 62.50% with an increasing trendline that was somewhat variable ranging from 55% to 72.50% (SD = 6.37%). Dan’s reaction time in the intervention phase had a mean of 2.89 s with a consistent, slightly ascending trendline ranging from 2.31 to 3.46 s (SD = 0.49 s). Between phases, two of the first three data points in the intervention phase were improved from the last three data points at baseline for both accuracy and reaction time. IRD for accuracy and reaction time were 60% and 40%, respectively, suggesting moderate improvement for accuracy and questionable improvement for reaction time between phases.

#### 3.4.2. Defense Recognition (T1–T2)

Dan’s mean accuracy score for T1–T2 items was 52% at baseline (SD = 8.82%) with variable data but a flat trendline. Dan’s mean accuracy score in the intervention phase was 67% (SD = 7.58%) with an ascending trendline. Dan’s mean reaction time at baseline was 4.64 s (SD = 1.96 s) with a decreasing trendline. Dan’s mean reaction time in the intervention phase was 3.04 s (SD = 0.34 s) with a slightly ascending trendline. IRD between baseline and intervention phases was 80% for accuracy and 62.50% for reaction time, indicating strong improvement for accuracy and moderate improvement for reaction time on questions related to identifying defensive schemes.

#### 3.4.3. Pass Selection (T3–T4)

On pass selection questions, Dan had a mean accuracy score of 49% (SD = 8.43%) at baseline with variable data but a flat trendline. In the intervention phase, Dan’s accuracy rose to 58% (SD = 9.75%) with an ascending trendline. Dan’s mean reaction time was 5.17 s (SD = 2.65 s) at baseline with a decreasing trendline. Dan’s mean reaction time in the intervention phase fell to 2.73 s (SD = 0.66 s) with a fairly flat trendline. IRD was 40% for accuracy and 60% for reaction time, indicating questionable improvement for accuracy and moderate improvement for reaction time. Cam correctly answered zero out of 16 adaptation sets at baseline (0%) and one out of 10 in intervention (10%). IRD for adaptation sets was 35%, indicating questionable improvement. Dan correctly answered one out of 20 adaptation sets at baseline (5%) and four out of 10 in intervention (40%). Interestingly, despite having the lowest WMC, Dan was the only participant to get both adaptation sets correct in a decision-making test, which occurred in Session 3 of the intervention phase. IRD for adaptation sets for Dan was 40%, indicating questionable improvement.

### 3.5. Social Validity

Participants filled out a social validity measure to assess their perceptions of the current intervention. As mentioned earlier, the measure was provided twice, once after completing the third learning module in the intervention phase, and again after completing the fifth module. This resulted in eight total responses to each item (two responses for each of the four participants). Complete response data for each item of the social validity measure can be found in Table 6.

In addition to comments completed on the social validity measures, participants mentioned several benefits that they perceived from the intervention during debriefing meetings, such as (a) the learning modules gave them a cognitive progression to follow, (b) the intervention helped them to direct their eyes to relevant visual cues rapidly, and (c) the players thought the learning modules could be helpful if a player was injured in order to get “mental reps.” Some suggestions for improvement included (a) having more than one “right” answer to a question, particularly with pass selection questions, and (b) organizing the learning modules by the opposing team rather than having different teams shown throughout, as was done for the intervention.

## 4. Discussion

The primary purpose of the current study was to test the efficacy of a novel perceptual training that utilized a modified temporal occlusion paradigm to improve high school quarterbacks’ tactical decision-making ability. There did not appear to be evidence of a causal relationship between changes in accuracy on the DMT and the introduction of the intervention, as outlined by the standards set forth by Kratochwill and colleagues [45]. However, there did appear to be moderate evidence for a causal relationship between the implementation of the intervention and an increase in speed of the participants’ decision-making reaction time. With regards to the first two research questions, there were similar results. The first research question asked how a temporal occlusion intervention may improve high school quarterbacks’ ability to recognize defensive schemes and the second question asked how the same intervention may impact high school quarterbacks’ pass selection. For questions related to identifying defenses (T1–T2) and selecting passes (T3–T4), there appeared to be no evidence for improvements in accuracy, but there was moderate evidence for improvement in reaction time after the intervention phase began. The third research question asked how a temporal occlusion intervention may impact high school quarterbacks’ ability to appropriately modify decisions, and there was no evidence that the intervention improved participants’ ability to do so. To assess for social validity, the fourth research question was concerned with the participants’ perceptions of the intervention. Participants reported believing the intervention was practical and effective as well as identified changes in their visual search behavior and cognitive processes as a result of completing the learning modules. Interestingly, the participant with the lowest WMC, Dan, showed evidence of improvement with regards to reaction time and accuracy. Dan’s improvement provides anecdotal support for Furley and Memmert’s [4] suggestion that coaches would be well advised to provide their athletes with sport-specific trainings, rather than traditional WM computer-based trainings, to improve tactical decision making. While these data are encouraging, limitations of the current study as well as implications for building off this initial research endeavor warrant discussion.

### 4.1. Limitations

The first potential limitation to the current study is the assessment measure used. Answers on the DMT were selected if the same two out of three experts agreed on all four decisions that had to be made in a play. Thus, at times, one expert chose a different response option for various decisions, which may have been appropriate to a situation, but was deemed “incorrect.” As Ward et al.’s [31] study demonstrated, it may be possible to have more than one appropriate response option to a situation in a complex sport task. A participant may have chosen an appropriate response option to an item, but it was categorized as incorrect during data analysis. Additionally, in the current study, expert feedback was used to strive for content validity. However, the content validity of the DMT could have been enhanced through obtaining a coefficient of content validity, such as Aiken’s *V* [48,49]. Therefore, more extensive assessment of the DMT and allowing for more than one “correct” answer (as suggested by one participant) may have improved the validity of the DMT.

Participant selection may have also been a threat to the current study [45]. For Aaron and Brett, there was a potential ceiling effect, because Aaron and Brett were able to get a large proportion of answers correct at baseline, leaving little room for growth. Given that the study utilized footage from the team’s prior football season, either Aaron or Brett were represented in the majority of the clips at the quarterback position, while Cam and Dan were not. Prior experience with the plays that were shown may have influenced Aaron and Brett’s decision-making ability as experiencing a play first-hand may have improved their pattern recognition and decision-making ability due to the meaningful experiences possibly being stored in their long-term memory [48]. Additionally, Aaron, Brett, and Cam all had substantially more quarterback experience than Dan. Dan had the least amount of quarterback experience and made clear improvements over the course of the intervention. Therefore, it may be the case that the current intervention is more appropriate for beginning quarterbacks rather than more experienced quarterbacks.

With regards to the context of the sessions, three participants completed the DMTs and LMs in study hall and one completed it after school (i.e., Cam). The decision to have three participants complete the sessions in study hall was due to logistical concerns (e.g., the lead author only had access to three laptops with the appropriate software, the meeting space was small, Cam was at a different school). Although participants were instructed not to discuss the DMTs or LMs in-session and the lead author monitored this, there was no way to monitor if the participants discussed the intervention outside of session. Additionally, completing the sessions as a group may have been more enjoyable to Aaron, Brett, and Dan as they could talk about topics not related to the study during their breaks, whereas Cam had to complete the DMTs and LMs by himself after school. It is possible Cam may have been more cognitively taxed than the other three participants when he completed the DMTs and LMs since they were administered after a full day of school. His accuracy did not improve over the course of the study and he was the only participant whose reaction time did not clearly improve. It is possible the cognitive toll of school contributed to him not benefitting as much from the intervention as he could have. Future research will want to account for such logistical concerns to more closely replicate the same context for each participant, improving experimental control and interpretation of results.

There is evidence that the intervention improved participants’ reaction time. However, a testing effect may have constrained the validity of the reaction time results. Three of the four participants had moderate to large improvements in their reaction time in all three forms of analysis (T1–T4, T1–T2, T3–T4) as measured by IRD. These results were promising and indicated that the intervention may have positively affected the speed of decision making of the quarterbacks included in this study. However, inspection of the various reaction time graphs reveals that the improvements may have been partially influenced by a practice/testing effect [45]. Future research that alternates phase repetitions for participants (e.g., ABAB designs) could mitigate this threat to the current study’s internal validity [45].

Finally, there was no significant change observed in participants’ ability to adapt their decisions on adaptation sets. Again, this was likely due to the assessment method. A low proportion of trials were chosen to be adaptation questions (0.20), in order to allow participants to rely primarily on automatic responses [4]. This low proportion did not allow for a wide range of data that could be inspected via visual data analysis (i.e., participants could only score 0, 1, or 2). Future single case research investigating quarterbacks’ abilities to adapt their decisions may benefit from using a strategy similar to Furley and Memmert’s [4]. In Furley and Memmert’s [4] study, participants were provided a prepotent response prior to watching a video clip, then asked to change or stick with that response depending on what they viewed. This strategy may allow researchers to collect more data regarding an individuals’ ability to change or stick with decisions appropriately, allowing for more data points to be examined during analyses.

### 4.2. Implications

More pilot testing is needed to create a temporal occlusion intervention for quarterbacks. Research using a more traditional, progressive temporal occlusion paradigm [17] may be beneficial to investigate when identifying defensive schemes is most accurate for experts. Once this moment is found, clips that occlude at the most appropriate moment can be incorporated into creating an assessment measure similar to the DMT used in this study. This same process can be utilized for identifying the most critical moment for pass selection. Allowing for more than one “correct” response option when it comes to pass selection may be appropriate. This could be achieved by experts listing what the relevant/appropriate response options are to a play when watching video clips, as was done in Ward et al.’s [31] study. Any option listed as an appropriate option by all experts could then be considered an appropriate response option [31]. Following the recommendations of participants, future trainings could also be organized by opposing team, rather than use different teams for each learning module, to further enhance what the experience would be like in a game situation for the athletes. Lastly, future researchers will want to obtain a coefficient of validity to improve content validity of a novel tactical decision-making assessment [49,50]. More thorough vetting in this manner may improve validity and reliability of the DMT (or similar novel decision-making assessments), which could be used to assess temporal occlusion interventions with quarterbacks.

Future perceptual trainings for quarterbacks could be improved through providing visual aids for participants. Participants appeared to understand the feedforward information their coaches provided them once they had answered a learning module question correctly and indicated that their visual search behavior had changed due to the guidance they had been provided. However, it is possible that learning could be improved, and the intervention could be applied more broadly (e.g., to individuals with less quarterback experience), if visual markers were included with the feedforward film clips to guide the participants’ gazes (e.g., [51]). How often participants re-watched clips that they answered correctly in the LMs was not recorded. Future research investigating how much participants re-watch previous clips could be valuable as experts are better able to recall patterns they have previously observed in their domain of expertise than novices (e.g., [52]), which may be due, in part, to the amount of practice and exposure they have had to their interest area [53,54]). Therefore, it may be that participants who re-watch clips more often may expedite their road to expertise, picking up on defensive patterns more quickly in the future and being more likely to make appropriate tactical decisions.

Dan’s improvement over the course of this study provides some anecdotal support for Furley and Memmert’s [4] suggestion that coaches wanting to improve the tactical decision making of their athletes would be better suited using sport-specific trainings than traditional computer-based WM trainings. Dan had the lowest WMC of all participants and yet was the only participant to get both adaptation sets correct in a trial of the DMT. Additionally, Dan demonstrated clear improvement in his accuracy and reaction time, indicating that the sport-specific training was helpful to his decision making. These data suggest that Dan’s WMC may not have constrained his capacity to improve his tactical decision making. Research further investigating this relationship between individuals’ WMC and tactical decision making in sport could help to provide coaches with practical interventions to improve their players’ tactical decision making.

### 4.3. Conclusions

The novel intervention used in this study was met with positive perceptions from participants and provided moderate evidence to support its efficacy in improving participants’ reaction time when recognizing defensive schemes and selecting appropriate passes. The use of computer-based perceptual trainings for quarterbacks in football is a valuable avenue for future investigation as it could lead to efficient, self-paced trainings that are both practical and beneficial to quarterbacks.

## Figures and Tables

**Table 1:** Demographic information and individual characteristics of participants.

Participant	Age	Race/Ethnicity	Class	Yrs. Playing Football	Yrs. Playing QB	Competitive Level	Hrs. Film per Week	Hrs./Wk Team Film In-Season	Hrs./Wk Team Film Out-Of-Season	Other Sports
Aaron	15	W	So.	9	6	Varsity/Junior Varsity	3–4	3–4	0–1	Golf
Brett	17	W	Jr.	6	5	Varsity	12–20	3–4	1	Baseball
Cam	14	MR	Incoming Fr.	8	7	N/A	1	1	0	Basketball, Baseball, Track-and-Field
Dan	15	B	Fr.	5	2	Freshman	4–5	4	1–2	Basketball

Note. W = White; MR = multi-racial; B = Black; Jr. = junior; So. = sophomore; Fr. = freshman; QB = quarterback; yrs. = years; hrs. = hours; wk = week.

**Table 2:** Schedule for participants when collecting baseline and intervention data.

Participant	Week 1		Week 2		Week 3		Week 4		Week 5	
	Session 1	Session 2	Session 3	Session 4	Session 5	Session 6	Session 7	Session 8	Session 9	Session 10
Aaron	B	B	I	I	I	I	I	X	X	X
Brett	B	B	B	I	I	I	--	I	I	X
Cam	B	B	B	B	I	I	I	I	I	X
Dan	B	B	B	B	B	I	I	I	I	I

Note. B = baseline phase; I = intervention phase; X = data collection complete; -- = participant was absent for session.

**Table 3:** Means and standard deviations for accuracy (%) and reaction time (s) on defense recognition (T1–T2) items.

Participant	Baseline				Intervention			
	Acc		RT		Acc			RT
	M	SD	M	SD	M	SD	M	SD
Aaron	80	7.07	3.79	1.08	93	5.70	2.66	0.46
Brett	84.17	2.04	1.96	0.47	85	0	1.38	0.11
Cam	71.88	5.30	2.10	0.53	68	5.70	2.16	0.38
Dan	52	8.82	4.64	1.96	67	7.58	3.04	0.34

**Table 4:** Means and standard deviations for accuracy (%) and reaction time (s) on pass selection (T3–T4) items.

Participant	Baseline				Intervention			
	Acc		RT		Acc		RT	
	M	SD	M	SD	M	SD	M	SD
Aaron	76.25	7.50	4.67	1.71	74	7.42	2.90	0.41
Brett	71.67	6.06	2.46	1.22	76	2.24	1.44	0.12
Cam	55.63	7.29	3.16	0.82	61	4.18	2.92	0.33
Dan	49	8.43	5.17	2.65	58	9.75	2.73	0.66

**Table 5:** Improvement rate difference (IRD) (%) on DMTs overall, defense recognition, pass selection, and adaptation sets.

Participant	T1–T4		T1–T2		T3–T4		Adaptation Sets
	Acc	RT	Acc	RT	Acc	RT	Acc
Aaron	60	80	60	55	−10	80	−60
Brett	33.33	100	16.67	100	20	80	23.33
Cam	22.50	37.50	−30	20	15	37.50	20
Dan	60	70	80	62.50	40	60	40

**Table 6:** Response percentages for each item and additional comments on the social validity scale.

Question	Strongly Disagree	Disagree	Agree	Strongly Agree
The video-based trainings have been beneficial to improving my decision making as a quarterback	--	--	12.50%	87.50%
I am able to keep my attention on the video-based trainings	--	--	100%	--
The video-based trainings interfere with my normal sport activities	87.50%	12.50%	--	--
The video-based trainings could cause a distraction for other players	75%	25%	--	--
I enjoy the video-based trainings we have done	--	--	25%	75%
Improving the accuracy of quarterbacks’ decision making is important	--	--	--	100%
Improving the speed of quarterbacks’ decision-making is important	--	--	--	100%
Additional Comments:	“I can really tell a difference in my decision making after these trainings and realizing the correct place to go with the ball. This really helps.” “I think it is cool and should be available to other positions.” “This helps!!” “The videos with feedback improve my decision making by a ton.” “I feel like these types of test could be helpful to other positions as well not just quarterbacks.”			

Note. There were eight responses total. Each participant completed the measure twice.
